# Temporal Structure in Audiovisual Sensory Selection

**DOI:** 10.1371/journal.pone.0040936

**Published:** 2012-07-19

**Authors:** Anne Kösem, Virginie van Wassenhove

**Affiliations:** 1 INSERM, U992, Cognitive Neuroimaging Unit, Gif/Yvette, France; 2 CEA, DSV/I2BM, NeuroSpin Center, Gif/Yvette, France; 3 Université Paris-Sud, Cognitive Neuroimaging Unit, Gif/Yvette, France; Bielefeld University, Germany

## Abstract

In natural environments, sensory information is embedded in temporally contiguous streams of events. This is typically the case when seeing and listening to a speaker or when engaged in scene analysis. In such contexts, two mechanisms are needed to single out and build a reliable representation of an event (or object): the temporal parsing of information and the selection of relevant information in the stream. It has previously been shown that rhythmic events naturally build temporal expectations that improve sensory processing at predictable points in time. Here, we asked to which extent temporal regularities can improve the detection and identification of events across sensory modalities. To do so, we used a dynamic visual conjunction search task accompanied by auditory cues synchronized or not with the color change of the target (horizontal or vertical bar). Sounds synchronized with the visual target improved search efficiency for temporal rates below 1.4 Hz but did not affect efficiency above that stimulation rate. Desynchronized auditory cues consistently impaired visual search below 3.3 Hz. Our results are interpreted in the context of the Dynamic Attending Theory: specifically, we suggest that a cognitive operation structures events in time irrespective of the sensory modality of input. Our results further support and specify recent neurophysiological findings by showing strong temporal selectivity for audiovisual integration in the auditory-driven improvement of visual search efficiency.

## Introduction

Many ecologically relevant events (such as speech, auditory and visual scenes, music…) present natural periodicities or statistical temporal regularities [1]–[4]. These temporal regularities provide useful cues to help parse and structure events out of complex sensory streams notably by building strong temporal expectations on the upcoming sensory inputs. For instance, it has previously been shown that a steady rhythmic presentation improves the detection of an event in a stream [5]–[7]: the detection of an auditory (visual) event is improved when it appears one period after the last auditory (visual) event, but impaired when it is presented earlier or later than at the instant predicted on the basis of the previous stimulation rate [5]–[7]. Such results have been interpreted in the context of the Dynamic Attending Theory (DAT) [8].

The DAT provides a mechanism for selective attention in time i.e. for the parsing of objects based on their inherent temporal structure or based on the temporal structure of an internal oscillator. One strong assumption of the DAT is that the brain can not only keep track of temporal regularities (or environmental rhythms) but also predict, on this basis, the arrival time of a transient event that fluctuates at the same rate. As such, the ‘temporal context’, defined as the relative timing between past AV events, becomes an important factor for attentional selection in time. One implementation of attentional selection in time heavily relies on oscillatory mechanisms that lock to the temporal structure of sensory events [8], [9] (Figure S001). This process can be compared to an expectancy profile that naturally allows attention to be engaged at the very point in time at which a stimulus is anticipated to appear or change [5]–[7]. This temporally precise allocation of attention could bear functional relevance for the early encoding and selection of features across sensory modalities. Thus, DAT sketches an attentional-tracking mechanism over time that is understudied yet offers interesting complementary views to more traditional space-, feature- or object-tracking approaches in the study of attention [10], [11]. Here, we asked whether the DAT could be extended across sensory modalities and whether temporal regularities can be shown to operate automatically in the selection of appropriate audiovisual (AV) events.

A first motivation for this experimental work is that the temporal structure of events is a well-known constraint for multisensory integration [12]–[19]; yet, previous studies have provided contradictory results regarding the automaticity of attentional selection for synchronous streams of AV stimuli. Using visual search paradigms with dynamic stimuli, the presence of rhythmic auditory stimuli synchronized with visual targets can either improve [20], [21] or have no effect [22] on visual search efficiency. One major difference in these studies was the rate at which AV events were displayed: no AV search efficiency was observed for 10 Hz [22] but improvements were reported for 1.1 Hz [20]. Second, recent neurophysiological findings have suggested that tracking the temporal structure of AV events likely operates in particular temporal regimes [23]–[25]. Neural oscillations are classically known to entrain to rhythmic stimuli [26], [27], thereby providing a direct mechanistic implementation for the DAT (Figure S1): neural entrainment modulates the excitability of tuned neural population *through time*. As such, the processing of events in phase with the entrained oscillation is facilitated due to higher neural excitability [28]. In AV context, it has been shown that rhythmic sounds lead to neural entrainment not only in auditory cortex but also in visual cortices [24]; this suggests that rhythmic auditory stimulation can modulate visual processing at relevant points in time and could presumably affect visual detection rates. Third, the AV modulation of neural excitability across sensory cortices has been demonstrated for neural oscillations of 1–2 Hz or “delta band” [23]–[25].The specific involvement of slow frequency oscillations (1–2 Hz range) may optimize early AV integration for stimuli in that dynamic range.

The temporal rates between studies [20] and [22], the known importance of AV transience for multisensory integration [21], [29]–[31], and the temporal structure of AV events [12], [17], [18] can all be limiting factors for automaticity in AV integration. Hence, we were interested in the effect of AV temporal rate and the temporal context it confers to visual search efficiency. Specifically, on neurophysiological grounds, the existence of a temporal threshold (1–2 Hz) on the automaticity of AV integration is here predicted.

**Figure 1:**
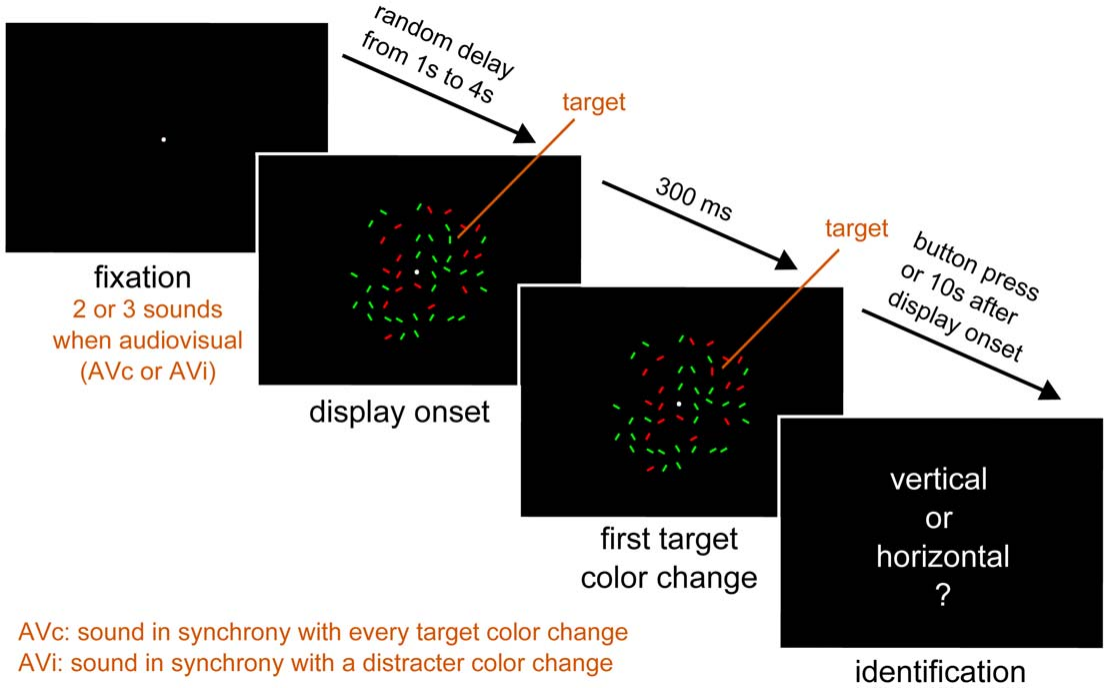
Experimental Paradigm. Each trial started with a fixation point lasting between 1 and 4 seconds (randomized across trials). In the audiovisual conditions (AVc, AVi) two or three sounds appeared before the visual display in order to avoid a surprise effect at the onset of the first sound. This was followed by the visual display with or without a sound (AVc and AVi or V, respectively). Participants were asked to find a horizontal or a vertical bar in the visual display while maintaining their gaze on the fixation point at all times. They were asked to answer as fast and as accurately as possible by pressing the space bar on the keyboard. One trial lasted a maximum of 10 seconds during which the participant was expected to have detected the target. After detection, participants were asked to identify the orientation of the detected target (vertical or horizontal). If the participant had not detected the target, he was nevertheless asked to make a guess. Therefore, this design allowed quantifying two dependent variables: reaction times (RTs – with a 10 sec imparted limit for the participant’s detection) and identification rate. In subsequent analysis, trials in which the target was not detected within 10 s were discarded for RTs. The experiment was run in 3 pseudo-randomized blocks corresponding to the display condition (V, AVc and AVi).

To test this hypothesis, we build on the visual conjunction search paradigm developed by van der Burg and colleagues [20]: a horizontal or vertical bar (visual target) surrounded by distracters of various orientations changed colors at particular temporal rates (Figure 1, Video S1). We used seven temporal rates and three set sizes to test whether the rate at which the visual target changed color alone (V), with a synchronized sound (AVc) or with a sound synchronized with a distracter (AVi) was a determining factor for search efficiency.

## Results

Visual search efficiency was quantified in terms of RTs and identification rate: for each trial, participants were asked to press a button as fast as possible when they saw the target; after detection, they reported its orientation in a 2-Alternative-Forced-Choice (2-AFC, “vertical” or “horizontal”) allowing the assessment of the correct identification rate. Statistical analysis was performed using a linear mixed effects model for RTs and a logistic regression model for identification rate [32], [33]. The fixed factors were display condition (3: AVc, AVi, and V), set size (continuous factor) and temporal rate (7 discrete levels). Subjects (n = 24) were a random effect. Significant fixed factors were assessed by means of a regression model simplification using the Akaike Information Criterion (AIC). Each model’s goodness of fit was evaluated against the inclusion of each variable and interactions across variables of interests. Table 1 summarizes the comparison of the obtained models. The main effects of ‘temporal rate’ and ‘set size’ and their interaction with the predictor ‘display condition’ accounted for a significant amount of variance on RTs and identification rate (Table 1: model 4 and 5, respectively). The interpretation of all models preceding model 5 and 4 are provided in text. The additional statistical analyses were conducted with the regression models containing all significant predictors and interactions for each dependent variable: namely, model 5 for RTs and model 4 identification rates. Specifically, all regression coefficients used to assess statistical significance (t-tests, Wald tests (yielding Z)) were directly drawn from these two models.

**Table 1:** Summary of linear mixed regression analyses.

RTs					
Regression models	Df model	AIC	ChisqChi	Df	Pr(>Chisq)
**model 1: display condition + (1|subject)**	5	55263			
**model 2∶1+ set size**	6	55243	22.92	1	1.69e-16***
**model 3∶2+ temporal rate**	12	55118	136.85	6	<2.2e-16***
model 4∶3+ display cond. *temporal rate	24	55090	51.43	12	7.84e-7***
model 5∶4+ display cond. *set size	26	55086	8.46	2	0.0015*
model 6∶5+ set size *temporal rate	32	55091	7.03	6	0.32
**IDENTIFICATION**					
**Regression models**	**Df Model**	**AIC**	**ChisqChi**	**Df**	**Pr(>Chisq)**
**model 1:display condition + (1|subject)**	4	8055.3			
**model 2∶1+ set size**	5	7965.9	91.3227	1	<2.2e-16***
**model 3∶2+ temporal rate**	11	7923.1	54.8172	6	5.047e-10***
model 4∶3+ display cond. *temporal rate	23	7875.9	71.2673	12	1.854e-10***
model 5∶4+ display cond. *set size	25	7877.8	2.0273	2	0.362
model 6∶5+ set size *temporal rate	31	7885.4	4.3897	6	0.624

Regression model minimization used the Akaike Information Criterion (AIC) and likelihood ratio. Three factors were analyzed: display condition (3 levels), set size (continuous factor) and temporal rate (7 discrete levels) plus one random effect (24: participants). Six models were tested to explain the data with increasing order of complexity, namely: model (1): the effect of display condition; model (2): model 1+ set size; model (3): model 2+ temporal rate; model (4): model 3+ display condition × set size; model (5): model 4+ display condition × temporal rate; model (6): model 5+ set size × temporal rate. Bold models designate those variables significantly contributing to model estimate.

### 1. Transient Sounds Affect Visual Search Efficiency Irrespective of Temporal Stimulation Rate

Participants were faster and more accurate at detecting the target in AVc than in V (significance of contrast coefficient AVc *vs.* V for RTs: t = 3.2, p<0.001; for identification: Z = −5.8, p<0.001) but slower and less accurate in detecting the target in AVi than in V (significance of contrast coefficient AVi *vs.* V for RTs: t = −5.9, p<0.001; for identification: Z = 8.5, p<0.001). An AV congruency effect (contrast AVc *vs.* AVi) was observed in both RTs and identification rates (RTs: t = 9.7, p<0.001; identification: Z = −13.3, p<0.001). These results suggest that a transient sound facilitates the detection of a synchronized visual target but also impairs target detection when it is synchronized with a distracter, in line with prior reports [20].

**Figure 2:**
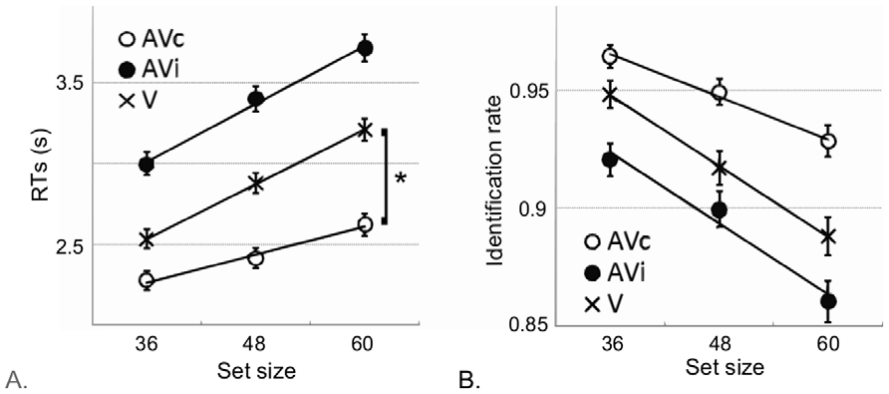
Effect of number of distracters on RT and identification rate collapsed over all temporal rates. Mean response times (A) and detection rates (B) per condition and per subject as a function of set size. Bars denote two SEM. A significant interaction was found between display condition and set size for RTs. The slope of the curve RTs  =  f(set size) was significantly lower in condition AVc than in condition AVi. The number of distracters affected the visual search less when a sound was synchronized with a target color change than in the absence of sound (V) or in desynchronized condition (AVi).

A main effect of set size was found (Figure 2; Table 1, “model 2: model 1+ set size”): across all display conditions, search efficiency decreases when the number of distracters increases (RT slope  = 7±2 ms/item, t = 4.3, p<0.001). As shown in Figure 2, the set size impaired RTs more in AVi than in AVc (AVi slope value: 11±3 ms/item; AVc slope value: 4±3 ms/item; t = 2.7, p<0.01). When gathering data across all temporal rates, no significant effect was found between the AVc and V slopes (V slope value 11±2 ms/item; t = 1.8, p = 0.058) or between the AVi and V slopes (t = −0.8, p = 0.4). Thus, the number of distracters influences the visual search less in AVc than in AVi or in V.

However, RTs cannot be taken as definite evidence for improvements in perceptual processing [34], [35]. No significant interaction between display condition and set size was observed for identification (Table 1) and the slopes for correct identification did not significantly differ across modalities when temporal rates were taken out of the model (Figure 2). Identification rates decreased with increasing number of distracters whereas the search remained most efficient in AVc and least efficient in AVi. According to our hypothesis, efficient AV search may not occur across all temporal rates (cf. main effects of temporal rates in RT and identification rate in Table 1, model 3) and we thus turn to the specific effects of temporal rate on visual search efficiency.

### 2. Temporal Rates and Attentional Selection

**Figure 3:**
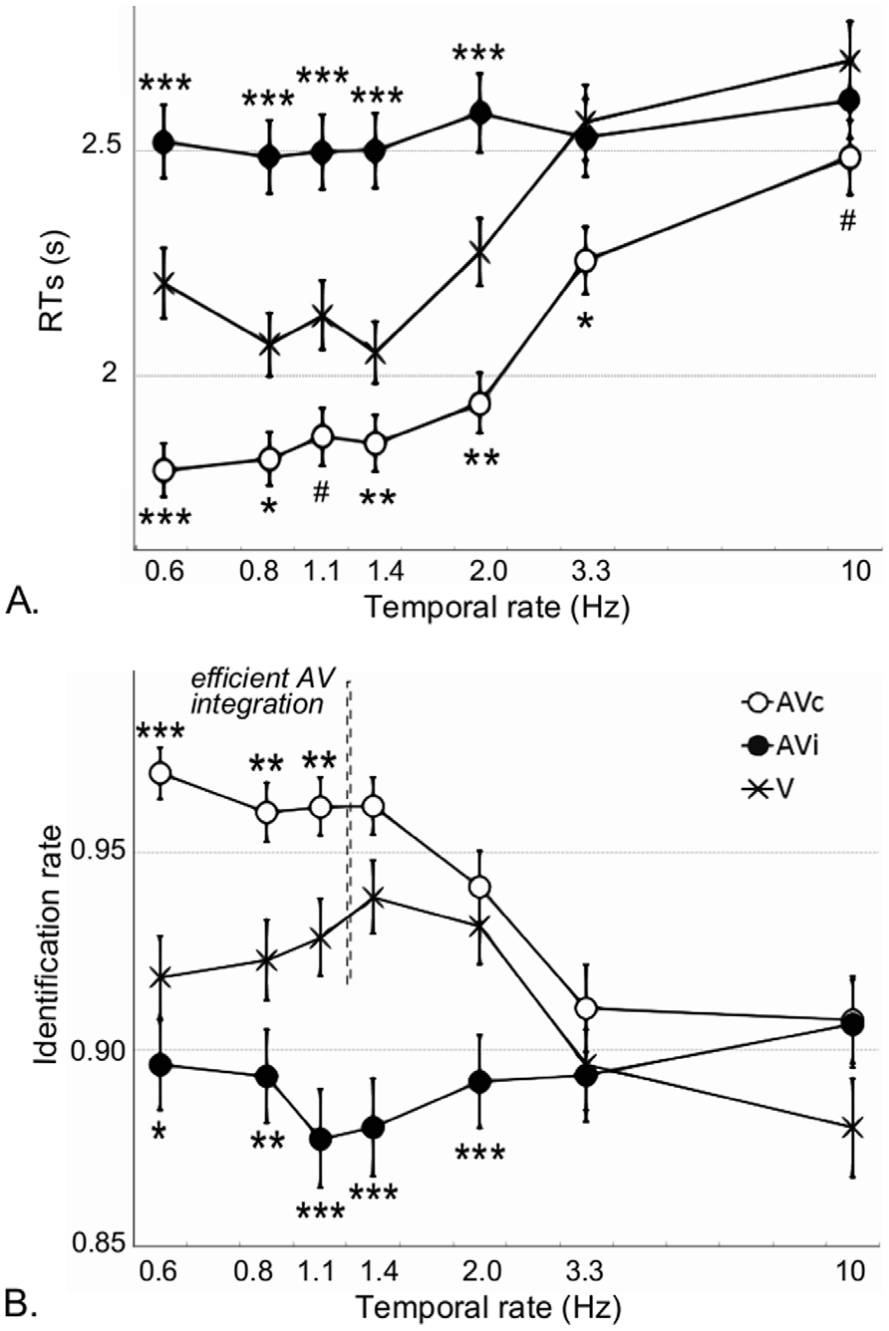
Main effect of temporal rates collapsed across all set sizes on reaction times and identification rate. Mean response times (A) and detection rates (B) per condition (V: crosses, AVc: filled circles and AVi: open circles). Bars denote are two SEM. A sound synchronized with the visual target color change fastens RTs for all temporal rates (Fig. 3a) and improves target detection only below 1.4 Hz (Fig. 3b). The level of significance between AV (AVc and AVi) and V conditions are reported as follows: *p<0.05; **p<0.01; ***p<0.001, ^#^p<0.055.

Temporal rates accounted for a significant amount of RT and identification rate variance (Table 1, model 3): significant effects were observed for both RT and identification rates between the different temporal rates (Tables S1 and S2, respectively). Overall, participants were faster and more accurate at temporal rates below 1.4 Hz compared to rates above 3.3 Hz irrespective of modality and set size (Figure 3, Table S1 and S2). Abrupt visual onsets are known to capture exogenous attention [36]. In this paradigm, as the temporal rate increases so does the number of color changes: this could lead to a larger temporal crowding effect in which individuating the visual target in time may become particularly challenging [37]. Interestingly, temporal rates significantly affected both RTs and identification rates in AVc and in V but not in AVi (Tables S3 and S4, respectively). The temporal margin introduced in our paradigm could have diminished the temporal crowding effect and benefited target identification in AVc but impaired it in AVi. However, the lack of temporal rate effect on the identification rate in AVi suggests that, consistent with the observed slower RTs, auditory information may either be disregarded or compete with desynchronized visual information (in this case, the target).

#### 2.1 Search efficiency and temporal stimulation rate

RTs were significantly faster in AVc than in V for almost all temporal rates (significance of contrast coefficients AVc *vs.* V at each temporal rate: t*_0.6 Hz_*  = 3.5, p<0.001; t*_0.8 Hz_*  = 2.4, p<0.05; t*_1.1 Hz_*  = 1.95, p = 0.05; t*_1.4 Hz_*  = 2.7, p<0.01; t*_2 Hz_*  = 3.2, p<0.01. t*_3.3 Hz_*  = 2.3, p<0.01. t*_10 Hz_*  = 1.95, p = 0.05) but slower in AVi compared to V only for temporal rates below 3.3 Hz (significance of contrast coefficients AVi *vs.* V at each temporal rate: t*_0.6 Hz_*  = −5.3, p<0.001; t*_0.8 Hz_*  = −5.8, p<0.001; t*_1.1 Hz_*  = −5.8, p<0.001; t*_1.4 Hz_*  = −4.2, p<0.001; t*_2 Hz_*  = 3.2, p<0.01. t*_3.3 Hz_*  = −1.3, ns; t*_10 Hz_*  = −1.1, ns). Note however that the limit of 3.3 Hz in AVi is not due to the interaction of RTs with temporal rate but rather to the slowing down of RTs in AVc and V with increasing temporal rates (cf. Fig. 3a). The constant RT difference (about 320 ms) across temporal rates between AVc and V support an effect of overall alertness affecting the central decision stage [38].

Identification was significantly worse in AVi compared to V, below 3.3 Hz (AVi *vs*. V: Z*_0.6Hz_*  = 2.5, p<0.05; Z*_0.8Hz_*  = 2.9, p<0.01; Z*_1.1Hz_*  = 3.8, p<0.001; Z*_1.4Hz_*  = 5.2, p<0.001; Z*_2Hz_*  = 4.3, p<0.001; Z*_3.3Hz_*  = 1.4, p = 0.15; Z*_10Hz_* <1); for temporal rates below 1.4 Hz, identification in AVc was significantly better than in V (AVc *vs* V: z*_0.6 Hz_*  = −3.5, p<0.001; Z*_0.8 Hz_*  = −3.3, p<0.01; Z*_1.1 Hz_*  = −2.7, p<0.01; Z*_1.4 Hz_*  = −1.8, p = 0.07; Z*_2 Hz_* <1; Z*_3.3 Hz_*  = −1.9, p = 0.055; Z*_10 Hz_*  = −1.4, p = 0.2).

These results suggest that true AV benefits in search efficiency (considering both RT and identification rate) are constrained to temporal rates below1.4 Hz: informational gain could be uniquely obtained in the range of temporal rates in which auditory information affects visual analysis and/or the robustness of the target representation and within which the temporal structure of events can be tracked.

#### 2.2 Two search regimes based on temporal rate

To establish whether AV search operates in two modes based on the temporal structure of events (namely, one of automatic AV integration at low temporal rates and one of AV competition at higher temporal rates), data were divided into two groups (temporal rates below and above 1.4 Hz; preliminary statistical analysis was conducted to determine this grouping albeit details are not reported here for sake of clarity).

**Figure 4:**
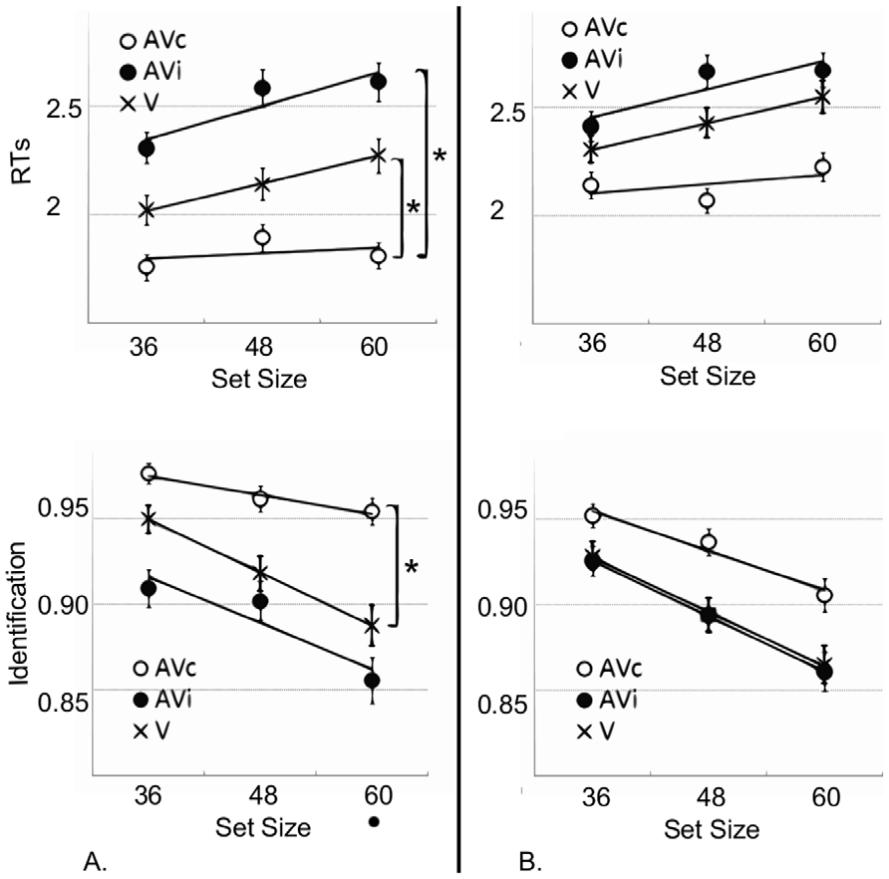
Grouped RTs and identification rates as a function of set size. Grouped RTs (upper panels) and identification rates (lower panels) as a function of set size for temporal rates under (A) and above (B) 1.4 Hz. Bars are two SEM. In the below 1.4 Hz group, slopes in AVc are shallower than in AVi and V conditions; this suggests that visual search is less impaired by distracters in AVc. In the above 1.4 Hz group, no significant differences in slopes were found.

A main effect of display condition on RTs was found for the below 1.4 Hz group (AVc *vs.* AVi: t = 14.77, p<0.001; AVc *vs.* V: t = 4.58, p<0.001; AVi *vs.* V: t = −9.99, p<0.001) and the above 1.4 Hz group (AVc *vs.* AVi: t = 11.54, p<0.001; AVc *vs.* V: t = 5.09, p<0.001; AVi *vs.* V: t = −6.27, p<0.001). Similarly, a main effect of display condition was found for identification rates in the below 1.4 Hz group (AVc *vs.* AVi: Z = −10.14, p<0.001; AVc *vs.* V: Z = −5.41, p<0.001; AVi *vs.* V: Z = 5.61, p<0.001) and in the above 1.4 Hz group (AVc *vs.* AVi: Z = −7.71, p<0.001; AVc *vs.* V: Z = −2.06, p<0.05; AVi *vs.* V: Z = 5.62, p<0.001). However, differences in identification rate between AVc *vs.* V and AVi *vs.* V conditions obtained in the below 1.4 Hz group were twice as large as those obtained in the above 1.4 Hz group (Figure 4).

More importantly, the effect size was only found to be significant for the below 1.4 Hz group between the conditions of interests (Figure 4), namely RTs and identification rates were less affected by the number of distracters in AVc than in V in this group (RT: AVc slope value: 1±3 ms/item, V slope value:11±3 ms/item; t = −2.1, p<0.05; identification AVc slope value: −0.1±0.02%/item, V slope value: −0.2±0.07%/item: Z = 2.1, p<0.05). The slope of RTs was also found to be much steeper in AVi than in AVc (AVi slope value: 13±4 ms/item;t = −2.0, p<0.05). Additionally, no difference in slopes was found between AVi and V (RTs: t = 0.3, ns, identification: Z = 0.2, ns). The major decrease in AVc slope compared to other conditions suggests that the target is more immune to the presence of visual distracters in this display condition; 1.4 Hz appears to be a temporal rate below which a synchronous sound automatically improves visual search efficiency by helping the individualization or segregation of visual targets presented in a dynamic stream of events.

## Discussion

In this study, we first replicated prior findings showing that RT and identification of a visual target in a dynamic conjunction search task is more efficient when a transient sound is synchronized with the visual target color change (AVc) at 1.1 Hz [20]. Additionally, we showed that visual search efficiency is impaired in AVi - sound synchronized with a visual distracter color change - compared to V or AVc. Crucially, we showed the existence of two temporal regimes: one in which AVc search reveals an automaticity profile for temporal rates below 1.4 Hz (no effect of number of distracters on RT or identification rate), and the other in which a competition profile is seen above 1.4 Hz.

The 1.4 Hz temporal limit found here may provide an insight on how to disambiguate conflicting results in the literature: whereas some studies have supported the existence of automaticity in AV integration [20], others have postulated that AV integration was post-attentional [22]. Our first working hypothesis was that both studies used a very different temporal rate (1.1 Hz in the former, ∼10 Hz in the latter). We now show that these temporal rates are indeed in and out of the efficiency search range observed here, respectively. Thus, our study suggests that the presence of transient events may be a necessary [21] but insufficient condition for automatic AV integration: specifically, we suggest that *shared temporal structure* between auditory and visual events matters.

### 1. AV Attentional Selection Depends on Temporal Structure

In this paradigm, two properties were shared between audition and vision: first, the transience of a sound aligned in time with the abrupt visual color change of the target or distracter and second, the temporal context, namely the relative temporal history between past AV events. One question is thus whether AV synchrony is a determining factor in sensory selection [20], [21] or whether the temporal structure of sensory events also matters for the observed improvements in AV search efficiency.

First, it has previously been argued that AV synchrony and the transience of events were critical factors for improved visual search efficiency [21]. If improved search efficiency solely relied on instantaneous AV integration (i.e. independently of the temporal context), the temporal stimulation rate effects could be argued to be a consequence of visual temporal crowding effects. Specifically, as the number of visual event changes increase (with faster temporal rate), visual search efficiency should decrease (slower RT, poorer identification rate). Such temporal crowding effect in vision should equally affect search efficiency irrespective of the modality of presentation (V, AVc, AVi). Our results clearly suggest that this is not the case: the profiles observed as a function of distracter number under different temporal rates indicate specificities both in the RT and in the identification rates with distinct patterns under and above 1.4 Hz.

Additionally, recent evidence [39] suggests that multisensory integration is pre-attentive when the spatial location of a visual stimulus is already resolved. The discrepancy between Alsius *et al.* [39] and van der Burg *et al.* [20] were deemed to rely on the nature of audiovisual stimuli: AV speech in the former case, transients AV events in the latter. If transient sounds can provide strong temporal anchors for the parsing of visual events thereby enhancing visual spatial search, temporal information extracted from auditory speech stream may be more subdued and less informative for visual segmentation [39]. In our experiment, transient tones were used (similar to [20]); it could be argued that at higher temporal rates, the search becomes inefficient due to the inability to extract temporal anchors from the acoustic stream. However, the fastest temporal rate in our experiment was 10 Hz (one 15 ms tone every 100 ms on average). Even at this high display rate, sounds are perceptually discrete and preserve the ability to affect in a non-random fashion the discrimination of temporal visual structure [12]. One interesting question is thus whether systematic manipulation of the salience in the natural temporal modulation of AV speech could affect the results reported in Alsius et al. [39].

Second, the temporal distance between visual events (target and distracters alike) was carefully controlled so that when a sound occurred, only one auditory and one visual event could be integrated at a time (see Methods). For most temporal rates tested here (except arguably 10 Hz, see Methods) the temporal distance was large enough for the visual target and the sound cue to integrate. If improvement in visual search efficiency solely relied on the integration of a single AV occurrence, no temporal rate effect should be observed. Hence, the significant temporal rate effects suggest that AV synchrony is not the sole factor in the sensory selection process: namely, the *temporal context* plays a critical role (in vision see also [40]–[42]).

The role of temporal context can further provide an account of the empirical discrepancies regarding the effect of a temporally incongruent sound in visual search. In our study, the sound-distracter pairing was temporally uncorrelated with the target’s color change, i.e. no temporal structure was shared between distracters and targets. In van der Burg *et al.* [20], the authors reported that a sound could improve visual search even when synchronized with a visual distracter: when a sound-distracter’s color change shortly preceded the target’s color change, efficient search was observed. However, in this control condition, the sound preserved its temporal cueing property with respect to the target: the sound-distracter pair and the target was systematically and on average separated by 200 ms. It is thus not entirely surprising that the sound-distracter pair kept on improving the target detection in [20] since temporal correlations between sound-distracter and target color changes were maintained.

For these reasons, we suggest that the improvement (impairment) observed in AVc (AVi) search efficiency below 1.4 Hz originates from AV perceptual grouping in time. One important issue in multisensory integration is whether the identity of AV information matters in the integration process. This has lead to two hypotheses: “multisensory enhancement hypothesis” or “perceptual grouping” hypothesis [29], [30]. Although our paradigm essentially addressed the former issue, our results are consistent with the ‘common-fate’ Gestalt principle, namely, features that have the same dynamics are more likely to be perceived as belonging to the same perceptual object. This has previously been demonstrated in visual [40]–[42] and in auditory grouping [43]–[44]. Our data add to this literature and suggest that a similar principle may be driving AV integration for certain temporal regimes. AV synchrony [20] but also, and crucially, AV temporal structure are fundamental to perceptual grouping in AV integration.

### 2. AV Temporal Prediction Benefits Visual Encoding

In line with - and as a multisensory extension of – the original DAT proposal [8] (Figure S1 panel A), selective attention can fluctuate in time and predict the arrival of future events based on the rate of presentation of the preceding stimuli. This, we suggest, may occur irrespective of the sensory modality of input. Additionally, the rhythmic occurrence of AV events may enable the elicitation of a (AV) temporal expectancy profile. It is noteworthy that two to three auditory events occurred before the visual display was shown thereby enabling a temporal expectancy profile to emerge even before the visual target first changed color. This may partly account for the overall faster RT observed in our study as compared to original findings [20].

Importantly, at rates below 1.4 Hz, both RTs and identification rates improved suggesting specificity in the AVc integration process. As observed within modalities [5]–[7], AV stimulus repetition may improve the precision of visual encoding. For instance, in agreement with the repetition-expectation effect [45], the extraction of visual information may be enhanced by the temporal predictability of the visual target based on the AVc temporal rate. Said differently, the shared temporal structure of AV events enables precise temporal prediction of the timing of the visual target change (Figure S1, panel B). In AVi condition, AV integration is prevented as sounds cue for the color change of a distracter (Figure S1, panel C). Additional experiments are needed to explore to which extent auditory and visual streams may enter in competition for attentional selection at these rates.

Like the DAT, alternative approaches based on the temporal statistics of events [46], [47] predict the establishment of an expectancy profile after the presentation of rhythmic stimuli. Based on the interval-based mechanism of perceptual timing [46], the extraction of temporal properties of a stimulus relies on the memory of interval durations between previous stimuli, not on the synchrony of events entrained by an internal oscillator. In our study, the arrival time of the next AV event would thus be computed based on the distribution of previous temporal delays between AV events. The central tendency and the dispersion of the distribution could encode the nature and the strength of the temporal expectancy. However, our main finding cannot be accounted for by these models: specifically, in case of repeated stimulation, such models predict an increase in strength and accuracy of the temporal prediction. In this paradigm, this would translate into a more efficient search for high temporal rates – considering that more events are displayed per second. The opposite effect was found here.

### 3. The Attentional Selection Threshold is Consistent with Neurophysiological Findings

Recent neurophysiological findings have suggested that attentional selection across sensory modalities may be implemented as entrainment of neural oscillations whether stimuli are rhythmic or present inherently a complex temporal structure [23]–[25], [28], [48]–[50]. Using simple rhythmic AV stimuli, the neural entrainment of auditory and visual cortices has been demonstrated in the 1–2 Hz range (“delta band”) [23]–[25].

One mechanistic view of brain function is that cortical oscillations naturally impose their temporal granularity on the parsing of sensory information. This has been shown in speech [51], in vision [48], [52] and extended to AV parsing [3]. If AV attentional selection operates in the 1–2 Hz range as suggested by monkey neurophysiology work [23]–[25], this mechanism should bear functional relevance to the central question of automaticity in AV integration [53]. These findings constitute a major prediction for the existence of a temporal boundary for AV attentional selection.

The 1.4 Hz limit in AV search efficiency is thus in line with neurophysiological predictions: specifically, neural entrainment above that temporal modulation would lead to a processing bottleneck of event tracking in time (Figure S1, panel B, cases illustrating the >1.4 Hz). Neural entrainment is characterized by an increased neural excitability at a particular phase of the entrained oscillation: if (i) neural entrainment is conceived as the mechanistic implementation of the expectancy profile hypothesized in the DAT [7] and (ii) auditory stimuli can entrain oscillations in visual cortices [24], then our results suggest that the encoding of visual events co-occurring with the sound will be more efficient at the time predicted by the auditory stimuli. In this context, the encoding of a visual event is as efficient for a target as for a distracter, as long as it shares its temporal structure with the auditory stream. Hence, when the visual event is a target, RTs and identification benefit from this automatic attention selection mechanism; when the visual event is a distracter, this mechanism impairs efficient detection of the target. In AVi, the automaticity of temporal parsing induced by the auditory rhythm hinders, and perhaps competes with, the detection of the visual stream that does not share the same temporal structure. Indeed, in AVi, the visual target stream cannot be tracked automatically and requires additional attentional resources as can readily be seen with the RTs increase and the lower identification rate irrespective of temporal rates (Figure 3). This attentional selection mechanism provides specific and testable neurophysiological predictions of increased (AVc) and decreased (AVi) search efficiency - or decreased and increased AV competition, respectively (Figure S1, panel B and C).

Interestingly, a complementary and very recent hypothesis has been put forward suggesting that auditory events affect the number and duration of visual fixations during visual search. Specifically, using a very similar paradigm, the authors showed that sounds synchronized with the visual target changes induced a ‘freezing’ of oculomotor scanning thereby allowing for a temporal and spatial enlargement of attentional focus [54]. These results also nicely suggest temporal limits on the attentional sampling mechanisms, albeit with an occulomotor perspective; the effect of different temporal rates on the occulomotor behavior could thus be interesting to explore. However, it is noteworthy that the notion of temporal freezing related to previous work [55] is difficult to reconcile with the faster RT in congruent AV conditions. Whether occulomotor scanning is implicated and necessary in the reported effects or whether a purely perceptual and neurophysiological temporal sampling account is sufficient remain to be elucidated.

One limitation remains in both our study and early neurophysiological findings [23]–[25], namely: the temporal structure of events is imposed by the stimulation rate and not necessarily imposed by ongoing neural oscillations. Automaticity is demonstrated in the context of salient entrainment of AV stimuli but it is unclear whether a similar limit on the automaticity of attentional selection would be observed for AV stimuli with more complex dynamics (e.g. as in AV speech [39]) or using a very different paradigmatic approach (e.g. as in [29]–[30]). Nevertheless, a recent behavioral study [56] using non-rhythmic stimulation further suggests that similar selection attentional mechanisms can affect perception: in a visual detection task, hit rates were shown to change periodically through time and maximum hit rates were phase-locked to the sound onset. The authors reported that the hit rates periodicity approximated 1 Hz although different temporal rates were not explicitly tested.

### 4. Conclusion

In a visual conjunction search paradigm, sounds can improve and impair search efficiency when synchronized or desynchronized with a visual target, respectively. Major improvements in search efficiency are limited to temporal stimulation rate slower than 1.4 Hz whereas impairments are consistent across temporal rates. Our results are interpreted in the context of the DAT [8] in the temporal frequency range predicted by monkey and human neurophysiology [23], [24], [25]: specifically, brain rhythms in the 1–2 Hz range naturally impose a limit on the attentional selection of events in time irrespective of sensory inputs. This can be considered a temporal Gestalt that operates at a slow rate across sensory modalities and enables automatic audiovisual integration.

## Materials and Methods

### 1. Subjects

Twenty-four volunteers (13 females, mean age: 22.5 years old) participated in the study. All had normal, corrected-to-normal vision, normal color vision and normal hearing, and were naive as to the purpose of the study. Each participant provided an informed consent in accordance with the Declaration of Helsinki (2008) and the Ethics Committee on Human Research at NeuroSpin (Gif-sur-Yvette, France).

### 2. Stimuli

Experiments were run in a darkened soundproof cabin. Participants were positioned on a headrest apparatus 70 cm away from a Viewsonic CRT monitor (19′′, 60 Hz). Auditory stimuli were presented via two speakers located on each side of the monitor. Visual stimuli consisted of an array of colored bars displayed on a black background (Figure 1). All bars were the same size (length: 0.57°; width: 0.19°) and randomly placed on a circular display with maximal eccentricity at 30°. All bars had random orientations except for the target which was vertical or horizontal. In each trial, the set size was 36, 48 or 60. A target could never appear within a radius of 3° around the white fixation point. In the initial frame, a color (red and green) was randomly assigned to each bar. All bars changed color through time. The timing of color changes was manipulated so that they always occurred at a given average temporal rate within one trial (but differed across trials). The temporal rates (F) tested were 0.56, 0.77, 1.1, 1.4, 2, 3.3 and 10 Hz. For a given trial presented at F, the delay between two color changes of a given bar was randomly chosen following a normal distribution with a mean of 1/F and a standard deviation of 1/4F. Three modalities of presentation were examined. In V, visual stimuli were displayed without any sound. In AV, a 15 ms (incl. 5 ms fade-in and -out) 2 kHz tone (44.1 kHz sample rate, 16 bit, mono) was synchronized with the color changes of a given bar in the display. In AVc, the sound was synchronized with the color change of the target; in AVi, the sound was synchronized with a randomly chosen distracter (the same one within a trial). Importantly, a sound had to be synchronized with only one bar at a time: to minimize the perceived synchrony between the color changes of the distracters and the sound, a temporal margin surrounding the sound/target onset was introduced during which no bars could change color. This temporal margin was scaled on the tested F: ±16.7 ms for 10 Hz, ±50.1 ms for 3.3 Hz, ±83.5 ms for 2.0 Hz, and ±117 ms for the remaining rates. In V and AVc, the temporal protection margin was applied to the target; in AVi, it was applied to the distracter.

### 3. Procedure

Participants were asked to find as fast and as accurately as possible the target while maintaining their gaze on a central fixation point. Each trial started with the presentation of the fixation point for a random duration (1–4 seconds) followed by the visual display. In AVc and AVi, two or three sounds were played before the visual onset to avoid surprise effects at the onset of the first sound. The presence of a sound synchronized with the target was expected to improve the speed of target detection [20]. In our paradigm, the sound onset was directly tied to F, namely, the higher the F, the earlier the auditory onset. To avoid a confounded faster RT, the first color change of the target occurred systematically at 300 ms after the display onset in all conditions. After detection or after 10 s has elapsed, participants reported the orientation of the target in a 2-Alternative-Forced-Choice (vertical or horizontal). The efficiency in visual search was quantified in terms of reaction times (RTs) and correct detection rate. We excluded the RTs from trials in which the target was not detected within 10 s (14% of the trials) from the data analysis. Each condition was repeated 15 times. The experiment was run in 3 blocks corresponding to the modality of presentation. Participants were told to ignore sounds as they were irrelevant to the task. The order of block presentation was counterbalanced across participants. The first block was used as a training session for all participants. The analysis focused on the last two blocks, when participants had reached asymptote on the task.

### 4. Statistical Analysis

Statistical analysis was performed using Linear Mixed Effects models [32] with R [57] (R Foundation for statistical computing). Linear mixed models can be thought of as a generalization of linear regression models: in mixed regression models, data are not aggregated, and statistics are made on all observations. Specifically, participants were considered as a random effect and separate regression models were fitted to the entire data set (i.e. one for each participant). This approach increases statistical power without over-fitting the data. On the contrary, classical regression models and repeated measures ANOVAs are based on the comparisons of measured means according to variables of interest (or fixed factors). Hence, unlike repeated measures ANOVAs in which comparisons are made between averaged data (information carried out by each observation is lost), in the mixed models used here, each observation is taken into account while considering the variability between subjects as a random effect. Additionally, the analysis of a categorical dependent variable (e.g. identification rate) is possible using a logistic mixed regression whereas ANOVAs may bring spurious results [33].

Thus, we selected this method as it is best suited for this study: fixed factors were display condition (3: AVc, AVi, and V), set size (continuous factor) and temporal rate (7 discrete levels). Subjects (n = 24) were a random effect. We considered ‘set size’ as a continuous factor because the RTs and identification rates as a function of set size fit well with the assumptions of a linear regression. We considered ‘temporal rate’ as a discrete factor because the dependency of RTs and identification rates as a function of temporal rate is not linear. Significant fixed factors can be assessed in two ways: (i) a regression model simplification using the Akaike Information Criterion (AIC) or (ii) the likelihood ratio using Chi square. The AIC is a measure that optimizes model fit by taking into account the amount of explained variance as well as the degrees of freedom. This procedure ensures that the obtained model achieves the best fit to the data with the minimum number of predictor variables. When two models are compared, the AIC provides information about whether the predictors added in the second model account for a significant amount of variance in the dependent variable. The best model corresponds to the minimal AIC. For instance, in the reported tables (e.g. Table 1), the list of models is provided along with their respective AIC index. The model that best fit the data is the one with the minimal AIC, here model 5 (for the RTs) and model 4 (for the identification rates). Consistent with this, the best models can also be found using Chi square.

The best model using the likelihood measure is defined by a significant Chi square test (Pr (>Chisq)) comparing one model in the list to the next (e.g.: model 1 *vs.* 2, then model 2 *vs.* 3 and so on). The last comparison providing a significant effect points to the best model: namely, in our example, model 5 (RTs) and 4 (identification rates). The “ChisqChi” value corresponds to twice the difference of the log likelihood of the two models. Both AIC and Chisqu values are reported in Table 1.

Simpler models (for instance, let’s consider models 1 to 4 for RTs in Table 1) do provide crucial information. Low AIC or significant Chi square tests for these models are interpreted as follows: the factor of interest (e.g. model 2, factor of set size) significantly impacts the model fit irrespective of all other factors – and hence, has a significant effect in our paradigm. This is analogous to stating a “main effect” for the more classic ANOVA approach. Here, the procedure is iterative such that adding another factor may enable better model fit (e.g. model 3 and so on) leading to the preferred model that explains most of the data (model 5 in our example). Hence, all factors up to model 5 (including their interactions) showed significant RTs effect.

The lme4 package [58] was used to obtain parameter estimates and the language package [32] was used to obtain the reported p-values. The ‘lmer’ function yielded regression coefficients and related t statistics (exclude degrees of freedom), and p-values were derived from a Markov Chain Monte Carlo (MCMC) [32]. Statistical tests were carried out on the contrast coefficients resulting from the selected linear mixed effect model. For instance, contrasting two levels for the display condition (AVc *vs.* V) yields a contrast coefficient submitted to a t-test for RTs and Wald test for identification rates. As a rule of thumb, statistical tests reported here vary according to the dependent variables, namely Student t-tests for the RTs and Wald tests for the identification rates [33].

## Supporting Information

Figure S1 **Dynamic Attending Theory (DAT), neural implementation as oscillatory entrainment and relevance for findings on AV selective attention.** **(A)** The DAT [8] postulates that attention is a dynamical process which oscillates in time and entrains to the temporal structure of events. **Event Timing:** dynamics of stimuli in a scene. Stimuli need not be isochronous – for illustrative purposes, events are represented with a particular rhythm. Events can be auditory or visual. **Modulation of attentional focus over time:** a temporal expectation profile builds up over time (i.e. after several occurrence of a same event) leading to a narrowing of attentional focus (from “wide” to “narrow”, [5]). The “narrow foci” are also times of high expectation (temporal prediction). Thus, the attentional profile oscillates between periods of high and low temporal expectation. **Implementation:** one suggested implementation of the DAT [5], [7] is via an oscillatory mechanism represented here as a simple waveform entrained to the rhythm of events. Recent neurophysiological evidence has suggested a similar neural implementation for attentional selection across auditory and visual sensory modalities, specifically with neural oscillations in the 1–2 Hz range [23]–[25]. In neural terms, high temporal expectations (or narrow attentional foci) are periods of high neural excitability. The encoding of events at the entrained rhythm is more efficient during period of increased neural excitability. For synchronized AV events, the auditory entrainment of oscillations in visual cortices leads to high expectation/excitability periods synchronized to the sound [23]–[25]. We now illustrate the implications for the AVc and AVi conditions tested in this study. (B) In AVc, the high expectation/excitability period is aligned to the target enabling faster RTs and improved identification rate. (C) In AVi, these periods are aligned with a distracter, leading to slower RTs and poorer identification rate. **Temporal rate effects:** modulation of visual search efficiency by the temporal rate of AV displays. The working hypothesis was that visual search efficiency would be frequency-specific: search would be efficient in the range of oscillatory entrainment but not above. Data revealed a 1.4 Hz boundary. Neural predictions: oscillations in visual cortex are entrained to the sound. Neural entrainment (alternation of high and low excitability phases) yields an expectancy profile favoring the encoding of visual events synchronized with the sounds (either the target (B), or one distracter (C)). When the temporal rate is above the oscillatory mechanism (B or C, right panels), the sound phase resets the entrained oscillation before it reaches a low excitability state. As the system is continuously solicited, no expectancy profile can be built and visual targets cannot benefit from the sound despite sharing the same temporal structure.
(TIF)

Table S1 Effect of temporal rate on RTs irrespective of display condition (V, AVc and AVi combined). Table shows contrast coefficients (*italics*, regression coefficients referring to contrast between two levels of one factor) between the different temporal rates and their related t values. Statistics were computed using mixed regression analysis with model 4 (cf. Table 1). Corrected p values were estimated using a Monte Carlo procedure. The reported significance values are as follows: *p<0.05; **p<0.01; ***p<0.001
(DOC)

Table S2 Effect of temporal rate on identification rate irrespective of display condition (V, AVc and AVi combined). Table shows contrast coefficients (*italics*, regression coefficients referring to contrast between two levels of one factor) between the different temporal rates and their related Z values (Wald tests). Statistics were computed using mixed regression analysis with model 4 (cf. Table 1). Corrected p values were estimated using a Monte Carlo procedure. The reported significance values are as follows: *p<0.05; **p<0.01; ***p<0.001.
(DOC)

Table S3 Effect of temporal rate on RTs per display condition. Table shows contrast coefficients between temporal rates for each display condition level and their related t values. Statistics were computed using mixed regression analysis with model 4 (cf. Table 1). Corrected p values were estimated using a Monte Carlo procedure. The reported significance values are as follows: *p<0.05; **p<0.01; ***p<0.001.
(DOC)

Table S4 Effect of temporal rate on identification rate per display condition. Table shows contrast coefficients between the temporal rates for each display condition and their related Z-values. Statistics were computed using mixed regression analysis with model 4 (cf. Table 1). Corrected p values were estimated using a Monte Carlo procedure. The reported significance values are as follows: *p<0.05; **p<0.01; ***p<0.001.
(DOC)

Video S1 **Experimental paradigm.** The video shows successively a trial (frequency of 1.1 Hz) in visual (V), congruent audiovisual (AVc), and incongruent audiovisual (AVi) conditions. Note that the display rate may be altered pending computer parameters.
(MPG)
