# Supplementary figures and images for: Temporal Structure in Audiovisual Sensory Selection
Source: PLoS One. 2012 Jul 19;7(7):e40936. doi: 10.1371/journal.pone.0040936 (PMC3400621; doi:10.1371/journal.pone.0040936)

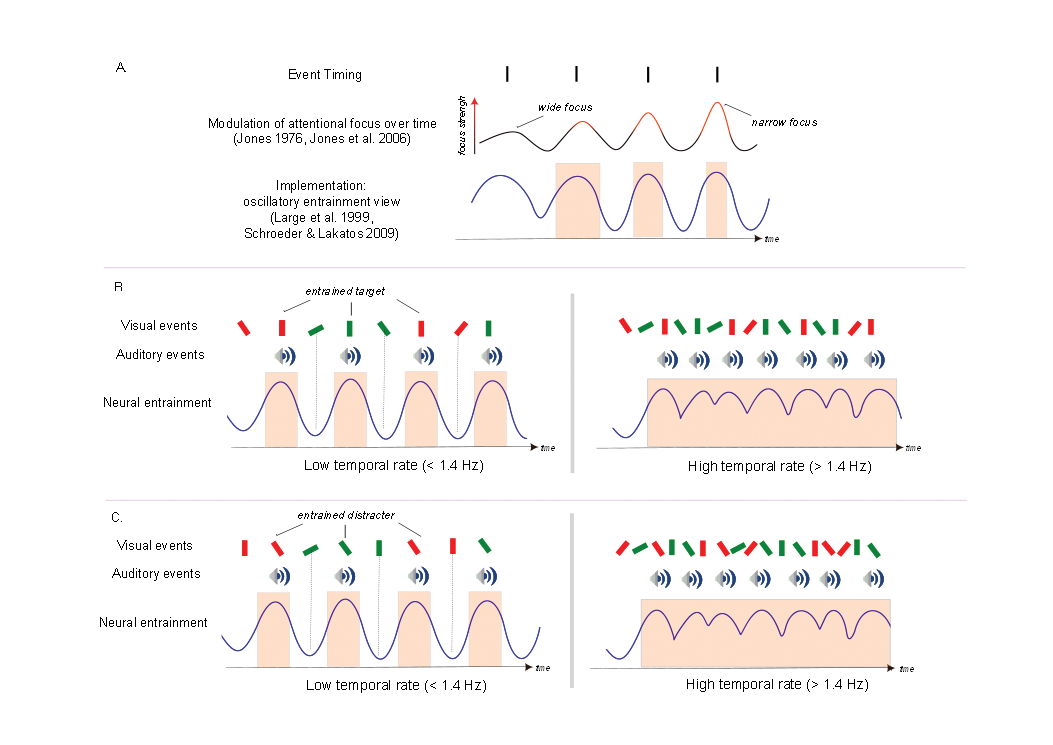

Supplement: Figure S1 — Dynamic Attending Theory (DAT), neural implementation as oscillatory entrainment and relevance for findings on AV selective attention. (A) The DAT [8] postulates that attention is a dynamical process which oscillates in time and entrains to the temporal structure of events. Event Timing: dynamics of stimuli in a scene. Stimuli need not be isochronous – for illustrative purposes, events are represented with a particular rhythm. Events can be auditory or visual. Modulation of attentional focus over time: a temporal expectation profile builds up over time (i.e. after several occurrence of a same event) leading to a narrowing of attentional focus (from “wide” to “narrow”, [5]). The “narrow foci” are also times of high expectation (temporal prediction). Thus, the attentional profile oscillates between periods of high and low temporal expectation. Implementation: one suggested implementation of the DAT [5], [7] is via an oscillatory mechanism represented here as a simple waveform entrained to the rhythm of events. Recent neurophysiological evidence has suggested a similar neural implementation for attentional selection across auditory and visual sensory modalities, specifically with neural oscillations in the 1–2 Hz range [23]–[25]. In neural terms, high temporal expectations (or narrow attentional foci) are periods of high neural excitability. The encoding of events at the entrained rhythm is more efficient during period of increased neural excitability. For synchronized AV events, the auditory entrainment of oscillations in visual cortices leads to high expectation/excitability periods synchronized to the sound [23]–[25]. We now illustrate the implications for the AVc and AVi conditions tested in this study. (B) In AVc, the high expectation/excitability period is aligned to the target enabling faster RTs and improved identification rate. (C) In AVi, these periods are aligned with a distracter, leading to slower RTs and poorer identification rate. Temporal rate effects: [file pone.0040936.s001.tif]
