# Supplementary material for: Temporal Structure in Audiovisual Sensory Selection
Source: PLoS One. 2012 Jul 19;7(7):e40936. doi: 10.1371/journal.pone.0040936 (PMC3400621; doi:10.1371/journal.pone.0040936)
Supplement: Table S1 — Effect of temporal rate on RTs irrespective of display condition (V, AVc and AVi combined). Table shows contrast coefficients (italics, regression coefficients referring to contrast between two levels of one factor) between the different temporal rates and their related t values. Statistics were computed using mixed regression analysis with model 4 (cf. Table 1). Corrected p values were estimated using a Monte Carlo procedure. The reported significance values are as follows: *p<0.05; **p<0.01; ***p<0.001 (DOC) [file pone.0040936.s002.doc]

| **RTs** | 0.8 Hz | 1.1 Hz | 1.4 Hz | 2.0 Hz | 3.3 Hz | 10 Hz |
| --- | --- | --- | --- | --- | --- | --- |
| 0.6 Hz |  |  |  |  |  |  |
| *contrast coefficient* | *-0.06* | *0.01* | *-0.04* | *0.1* | *0.31* | *0.47* |
| t value, significance | -1 ns | 0.2 ns | -0.7 ns | 1.7 ns | 5.7 *** | 8.0 *** |
| 0.8 Hz |  | 0.07 | 0.02 | 0.16 | 0.39 | 0.54 |
|  |  | 1.2 ns | 0.3 ns | 2.7 * | 6.6 *** | 8.9 *** |
| 1.1 Hz |  |  | *-0.05* | *0.09* | *0.33* | *0.47* |
|  |  |  | -0.8 ns | 1.5 ns | 5.4 *** | 7.7 *** |
| 1.4 Hz |  |  |  | *0.14* | *0.38* | *0.52* |
|  |  |  |  | 2.3 * | 6.2 *** | 8.5 *** |
| 2.0 Hz |  |  |  |  | *0.24* | *0.38* |
|  |  |  |  |  | 3.8 *** | 6.1 *** |
| 3.3 Hz |  |  |  |  |  | *0.14* |
|  |  |  |  |  |  | 2.2 * |
