# Supplementary material for: Temporal Structure in Audiovisual Sensory Selection
Source: PLoS One. 2012 Jul 19;7(7):e40936. doi: 10.1371/journal.pone.0040936 (PMC3400621; doi:10.1371/journal.pone.0040936)
Supplement: Table S3 — Effect of temporal rate on RTs per display condition. Table shows contrast coefficients between temporal rates for each display condition level and their related t values. Statistics were computed using mixed regression analysis with model 4 (cf. Table 1). Corrected p values were estimated using a Monte Carlo procedure. The reported significance values are as follows: *p<0.05; **p<0.01; ***p<0.001. (DOC) [file pone.0040936.s004.doc]

| **AVc** | **0.8 Hz** | **1.1 Hz** | **1.4 Hz** | **2.0 Hz** | **3.3 Hz** | **10 Hz** |
| --- | --- | --- | --- | --- | --- | --- |
| 0.6 Hz | *-0.02* | *0.06* | *0.01* | *0.13* | *0.54* | *0.77* |
|  | -0.3 ns | 0.7 ns | 0.1 ns | 1.4 ns | 5 .7 *** | 8.2 *** |
| 0.8 Hz |  | *0.09* | *0.04* | *0.15* | *0.56* | *0.79* |
|  |  | 1.0 ns | 0.4 ns | 1.6 ns | 6.0 *** | 8.4 *** |
| 1.1 Hz |  |  | *-0.05* | *0.07* | *0.48* | *0.71* |
|  |  |  | -0.5 ns | 0.7 ns | 4.9 *** | 7.3 *** |
| 1.4 Hz |  |  |  | *0.12* | *0.53* | *0.76* |
|  |  |  |  | 1.2 ns | 5.4 *** | 7.7 *** |
| 2.0 Hz |  |  |  |  | *0.41* | *0.64* |
|  |  |  |  |  | 4.1 *** | 6.4 *** |
| 3.3 Hz |  |  |  |  |  | *0.23* |
|  |  |  |  |  |  | 2.3 * |
| **AVi** | **0.8 Hz** | **1.1 Hz** | **1.4 Hz** | **2.0 Hz** | **3.3 Hz** | **10 Hz** |
| 0.6 Hz | -0.07 | -0.02 | -0.004 | 0.06 | 0.01 | 0.08 |
|  | -0.6 ns | -0.1 ns | -0.1 ns | 0.5 ns | 0.1 ns | 0.7 ns |
| 0.8 Hz |  | 0.06 | 006 | 0.13 | 0.08 | 0.15 |
|  |  | 0.1 ns | 0.6 ns | 1.1 ns | 0.7 ns | 1.4 ns |
| 1.1 Hz |  |  | 0.01 | 0.08 | 0.03 | 0.10 |
|  |  |  | 0.1 ns | 0.6 ns | 0.2 ns | 0.8 ns |
| 1.4 Hz |  |  |  | 0.06 | 0.02 | 0.08 |
|  |  |  |  | 0.5 ns | 0.2 ns | 0.8 ns |
| 2.0 Hz |  |  |  |  | -0.05 | 0.02 |
|  |  |  |  |  | -0.4 ns | 0.2 ns |
| 3.3 Hz |  |  |  |  |  | 0.07 |
|  |  |  |  |  |  | 0.6 ns |
| **V** | **0.8 Hz** | **1.1 Hz** | **1.4 Hz** | **2.0 Hz** | **3.3 Hz** | **10 Hz** |
| 0.6 Hz | -0.08 | 0.01 | -0.11 | 0.11 | 0.43 | 0.56 |
|  | -0.7 ns | -0.1 ns | -1.1 ns | 1.2 ns | 4.4 *** | 5.6 *** |
| 0.8 Hz |  | 0.07 | -0.03 | 0.20 | 0.51 | 0.64 |
|  |  | 0.8 ns | -0.3 ns | 2.0 * | 5.2 *** | 6.3 *** |
| 1.1 Hz |  |  | -0.11 | 0.12 | 0.44 | 0.56 |
|  |  |  | -1.1 ns | 1.2 ns | 4.4 *** | 5.5 *** |
| 1.4 Hz |  |  |  | 0.23 | 0.55 | 0.67 |
|  |  |  |  | 2.3 * | 5.5 *** | 6.6 *** |
| 2.0 Hz |  |  |  |  | 0.33 | 0.45 |
|  |  |  |  |  | 3.1 ** | 4.2 *** |
| 3.3 Hz |  |  |  |  |  | 0.12 |
|  |  |  |  |  |  | 1.1 ns |
