# Supplementary material for: Temporal Structure in Audiovisual Sensory Selection
Source: PLoS One. 2012 Jul 19;7(7):e40936. doi: 10.1371/journal.pone.0040936 (PMC3400621; doi:10.1371/journal.pone.0040936)
Supplement: Table S4 — Effect of temporal rate on identification rate per display condition. Table shows contrast coefficients between the temporal rates for each display condition and their related Z-values. Statistics were computed using mixed regression analysis with model 4 (cf. Table 1). Corrected p values were estimated using a Monte Carlo procedure. The reported significance values are as follows: *p<0.05; **p<0.01; ***p<0.001. (DOC) [file pone.0040936.s005.doc]

| **AVc** | **0.8 Hz** | **1.1 Hz** | | **1.4 Hz** | | **2.0 Hz** | | **3.3 Hz** | | **10 Hz** | |
| --- | --- | --- | --- | --- | --- | --- | --- | --- | --- | --- | --- |
| 0.6 Hz | *-0.13* | *-0.24* | | *-0.38* | | *-0.72* | | *-1.28* | | *-1.40* | |
|  | -0.84 ns | -0.97 ns | | -1.71 ns | | -3.11 ** | | -5.25 *** | | -5.82 *** | |
| 0.8 Hz |  | *-0.11* | | *-0.25* | | *-0.59* | | *-1.15* | | *-1.27* | |
|  |  | -0. 13 ns | | 0.89 ns | | -2.34 * | | -4.61 *** | | -5.23 *** | |
| 1.1 Hz |  |  | | *-0.19* | | *-0.54* | | *-1.03* | | *-1.16* | |
|  |  |  | | -0.76 ns | | -2.23 * | | -4.52 *** | | -5.14 *** | |
| 1.4 Hz |  |  | |  | | *-0.34* | | *-0.84* | | *-0.97* | |
|  |  |  | |  | | -1.49 ns | | -3.89 *** | | -4.52 *** | |
| 2.0 Hz |  |  | |  | |  | | *-0.50* | | *-0.62* | |
|  |  |  | |  | |  | | -2.46 * | | -3.15 ** | |
| 3.3 Hz |  |  | |  | |  | |  | | *-0.13* | |
|  |  |  | |  | |  | |  | | -0.71 ns | |
| **AVi** | **0.8 Hz** | **1.1 Hz** | | **1.4 Hz** | | **2.0 Hz** | | **3.3 Hz** | | **10 Hz** | |
| 0.6 Hz | *-0.03* | *-0.20* | | *-0.17* | | *-0.05* | | *-0.03* | | *0.14* | |
|  | -0.18 ns | -1.12 ns | | -0.98 ns | | -0.26 ns | | -0.16 ns | | 0.73 ns | |
| 0.8 Hz |  | *-0.16* | | *-0.14* | | *-0.01* | | *0* | | *0.17* | |
|  |  | -0.94 ns | | -0.80 ns | | -0.08 ns | | 0.01 ns | | 0.91 ns | |
| 1.1 Hz |  |  | | *0.02* | | *0.15* | | *0.17* | | *0.33* | |
|  |  |  | | 0.14 ns | | 0.86 ns | | 0.95 ns | | 1.90 ns | |
| 1.4 Hz |  |  | |  | | *0.13* | | *0.14* | | *0.31* | |
|  |  |  | |  | | 0.72 ns | | 0.81 ns | | 1.70 ns | |
| 2.0 Hz |  |  | |  | |  | | *0.02* | | *0.18* | |
|  |  |  | |  | |  | | 0.09 ns | | 0.99 ns | |
| 3.3 Hz |  |  | |  | |  | |  | | *0.17* | |
|  |  |  | |  | |  | |  | | 0.89 ns | |
| **V** | **0.8 Hz** | **1.1 Hz** | **1.4 Hz** | | **2.0 Hz** | | **3.3 Hz** | | **10 Hz** | |  |
| 0.6 Hz | *-0.06* | *0.02* | *0.13* | | *0.11* | | *-0.63* | | *-0.73* | |  |
|  | -0.28ns | 0.16 ns | 0.64 ns | | 0.55 ns | | -3.36 *** | | -3.94 *** | |  |
| 0.8 Hz |  | *0.08* | *0.19* | | *0.17* | | *-0.58* | | *-0.68* | |  |
|  |  | 0.40 ns | 0.93 ns | | 0.83 ns | | -3.13 ** | | -3.72 *** | |  |
| 1.1 Hz |  |  | *0.11* | | *0.09* | | *-0.67* | | *-0.77* | |  |
|  |  |  | 0.53 ns | | 0.43 ns | | -3.52 *** | | -4.11 *** | |  |
| 1.4 Hz |  |  |  | | *-0.02* | | *-0.78* | | *-0.89* | |  |
|  |  |  |  | | -0.09 ns | | -4.03 *** | | -4.61 *** | |  |
| 2.0 Hz |  |  |  | |  | | *-0.76* | | *-0.86* | |  |
|  |  |  |  | |  | | -3.93 *** | | -4.51 *** | |  |
| 3.3 Hz |  |  |  | |  | |  | | *-0.10* | |  |
|  |  |  |  | |  | |  | | -0.63ns | |  |
